# Impact of personality on cadet academic and military performance within mediating role of self-efficacy

**DOI:** 10.3389/fpsyg.2023.1266236

**Published:** 2023-10-16

**Authors:** Svajone Bekesiene

**Affiliations:** General Jonas Zemaitis Military Academy of Lithuania, Vilnius, Lithuania

**Keywords:** personality traits, self-efficacy, academic performance, military performance, cadets, mediation

## Abstract

**Background:**

The current operational military environment is changing, complex, unpredictable, and ambiguous. Due to such situations, soldiers are constantly forced to think about their values, norms, and roles that should be part of their profession. Consequently, they must first be educated and trained on how to behave in a particular operational military environment. Pursuing an officer’s education at military academies is very difficult not only physically but also psychologically. Cadets are required to be prepared to lead in extreme environments upon graduation. Despite the fact that military tasks are technically complex, the individual operational activities of soldiers are gaining more and more strategic meaning. Therefore, the importance of selecting the process and military education programs of soldiers is increasingly stressed. Cognitive abilities and skills individually predict performance in academic and professional settings, but it is less clear how personality can influence performance. Therefore, this study focused on the explanation of the individual factors that affect the achievements of the cadets. Specifically, the objective of this study was to examine direct and mediated relationships between personality traits and the military and academic performance of cadets.

**Methods:**

This study followed a quantitative method analysis. The research models were assessed using the structural equation modeling technique. Bootstrap was applied to evaluate a 95% level confidence interval on estimates with 5,000 bootstrap samples, and to evaluate direct and indirect effects. The analysis was based on a sample of 120 cadets from the Lithuanian Military Academy. The effects on military and academic performance were evaluated using the Self-Efficacy scale, the Big Five personality trait scale, academic performance was evaluated through academic grades and military performance was evaluated using instructor ratings.

**Results:**

To support our hypotheses, it was found that self-efficacy has a mediating effect on the performance of cadets. Additionally, the traits of conscientiousness, openness to experience and extraversion were related to both military and academic performance. Furthermore, self-efficacy appeared as a partial mediator of the relationship between personality traits and cadet performance.

**Conclusion:**

The findings of this study help clarify the relationship between the personality traits of the cadets and the military and academic performance. In addition, these results may be useful for the further development of military education and training, for the development of testing, and selection of military personnel.

## 1. Introduction

The current operational military environment is changing, complex, unpredictable, and ambiguous (Boe et al., 2017). This is the reason why the armed forces of various countries pay serious attention to their selection and training of military personnel (Haralambie, 2016; Rodden-Aubut and Tracey, 2022). Despite the fact that successful performance of warriors has always been one of the main challenges of the armed forces (Sellman et al., 2010), the individual effective actions of soldiers are becoming more and more significant (Shamir and Ben-Ari, 2018). Military professionals, including soldiers and officers, are expected to meet high standards that can help them deal with difficult situations. Therefore, self-efficacy, which refers to an individual’s belief in their ability to complete tasks, overcome obstacles, and succeed in specific situations, plays a critical role in the ability of soldiers to deal effectively with unexpected circumstances (Boe et al., 2018). According to social cognitive theories (Bandura, 1997) academic self-concept and self-efficacy can play a significant role in determining one’s motivation, perseverance, and resilience (Djourova et al., 2020; Bekesiene et al., 2022; Kanapeckaitė et al., 2022; Bekesiene et al., 2023). As military professionals often encounter high-stress situations that require quick decision making and effective problem-solving (Myrseth et al., 2018; Smaliukiene et al., 2022), the strong self-efficacy helps them manage stress by improving their belief in their ability to cope with and overcome challenges. This, in turn, can reduce anxiety, increase focus, and improve decision-making under pressure. Resilience in the military is also crucial to bounce back from setbacks, adapt to new situations, and maintain overall well-being (Harms et al., 2018; Dimas et al., 2021). Individuals with high self-efficacy are more likely to view setbacks as temporary obstacles that can be overcome with effort and determination. This mindset enables them to bounce back from failures, learn from their experiences, and persist in achieving their goals (Nindl et al., 2018).

Soldiers and officers with high levels of self-efficacy are more likely to undertake challenging tasks, persist in the face of adversity, and adapt to changing circumstances (Myrseth et al., 2018). Self-efficacious individuals tend to approach tasks with confidence and a belief in their ability to succeed. This mindset positively influences people’s performance, as they are more likely to set challenging goals and exert the necessary effort to achieve them. Thus, an individual’s ability to develop a high level of self-efficacy becomes an important factor to contribute to the acquisition of specific military skills and capabilities (Johansen et al., 2013; Boe et al., 2018).

The five Big Five traits that characterize the five dimensions of personality (openness to experience, conscientiousness, extraversion, agreeableness, and neuroticism) typically refer to enduring patterns of thoughts, feelings, and behaviors that characterize an individual character have been widely studied in psychology (Costa and McCrae, 1999; John and Srivastava, 1999). Scholars used the Five-Factor Model (FFM) to explain how the essential character of a person affects the academic achievements of the individuals (Bahçekapili and Karaman, 2020; Mammadov, 2022). Considering personality as a complex system and seeking to improve leader selection and development programs for United States military academy cadets, these five personality dimensions were used to evaluate leader performance (Bartone et al., 2009). Additionally, previous studies have acknowledged that both self-efficacy beliefs and personality traits can help predict academic performance (Caprara et al., 2011; De Feyter et al., 2012; Zuffianò et al., 2013; Buch et al., 2015; Fosse et al., 2015). Personality traits and self-efficacy affect people’s behavior in a different way (Bandura, 1997). So, the scholars used self-efficacy as a mediator to explain the interaction of the person’s behavior with the environment and proved the influence of personality traits through self-efficacy (Caprara et al., 2011; Bahçekapili and Karaman, 2020).

To understand the complex processes that link the personality of cadets and the military performance, the primary objective of this study was to test the mediation effects of self-efficacy between the Big Five traits and military performance. Previous studies focused mainly on the evaluation of student beliefs and attitudes toward their ability to achieve academic success by using self-efficacy mediation to academic performance in civil universities (Alyami et al., 2017; Doménech-Betoret et al., 2017; Li et al., 2022). As the existing literature provides limited information on the effect of personality traits on military performance, this study is aimed at evaluating the military performance of cadets. Taking into account the fact that in military academies, cadets receive not only military education, their academic achievements were also evaluated. Specifically, the objective of this study was to examine the direct and indirect pathways linking personality traits and self-efficacy. Therefore, the direct pathway analysis was used to assess the direct effects of the Big Five on self-efficacy, academic, and military performance separately, while the indirect pathway analysis assessed the mediation effects of self-efficacy on five personality dimensions to military and academic performance.

## 2. Theoretical background and hypotheses

### 2.1. Direct effect of personality traits to self-efficacy, academic and military performance

The Big Five model that represents the five dimensions of individual behaviors includes extraversion, agreeableness, conscientiousness, emotional stability (neuroticism), and openness to experience (intellect of personality) have attracted the attention of researchers. A literature review showed that personality traits are important in that they define an individual’s tendencies toward certain behaviors in a wide range of functioning (Hoyle, 2006). Also, according to previous research (Poropat, 2009) there was proven that conscientiousness and openness can show stable associations with academic achievement in comparison to other personality traits (Poropat, 2014). Conscientiousness is determined by personal qualities such as responsibility, the ability to plan, organize, and achieve. Additionally, conscientiousness is associated with methodical and analytical learning (Miller et al., 1999; Eisenberg et al., 2014; Roberts et al., 2014). Meanwhile, it was proved that the openness to experience personality trait may reflect an individual’s positive attitude toward the complex learning process and experience (Matz, 2021), and the pursuit of knowledge that is the result of consistent learning (Komarraju et al., 2009; DeYoung, 2015).

According to the theoretical background, academic performance refers to the level of achievement or success that cadets attain in their academic pursuits, such as coursework, exams, projects, and overall grades. It reflects their ability to grasp and apply knowledge, meet academic requirements, and demonstrate their understanding of the subjects they are studying. Achieving academic success can boost students’ self-confidence and belief in their abilities. When students perform well academically, they develop a sense of competence and a belief that they can tackle new challenges, experience a sense of accomplishment, and reduced anxiety about their future prospects (Pascarella and Terenzini, 2005). It is important to note that academic success should not be the sole measure of a student’s worth or potential. Each student is unique, and success can be defined in various ways beyond academic achievements (Ransdell, 2001). Various studies have explored the relationship between personality traits and academic performance, and some consistent findings have emerged. The results of the Hakimi et al. (2011) study agreed that conscientiousness was the most significant predictor variable, which enlightened 39 percent of variance in academic achievement, but gender differences in personality characteristics and academic achievement were insignificant; Mateus et al. (2021) identified a correlation between personality traits related to cognitive functioning and academic performance; Mammadov (2022) found that academic performance could be explained by 27.8 percent of the effects of cognitive ability and personality traits, and conscientiousness appeared as a strong and robust predictor of academic performance.

A literature review specified that performance in the military context and personality traits may also be linked. But only certain traits have been found to be particularly relevant and can impact an individual’s effectiveness in military roles. Bobdey et al. (2021) explored the correlation between personality traits of cadets undergoing training at an Armed Forces Training Academy and their performance in terms of their military and academic pursuits. Bobdey et al. (2021) results showed that cadets with high personality traits scores in neuroticism and low scores in conscientiousness had performed poorly in all domains for performance evaluation. Additionally, the conscientiousness facet was found to be positively correlated with performance in academic and military tasks (Fosse et al., 2015).

Similarly, individual’s performance was found in close association with person’s self-efficacy. Self-efficacious individuals tend to approach tasks with confidence and a belief in their ability to succeed (Schunk and Pajares, 2009). This mindset positively influences their performance, as they are more likely to set challenging goals and exert the necessary effort to achieve them. The concept of self-efficacy originated from social cognitive theory, developed by the psychologist Albert Bandura (1997). According to Bandura (1997), self-efficacy plays a central role in motivation, behavior, and personal development. Bandura’s social cognitive theory emphasizes the role of cognitive processes, observational learning, and social interaction in shaping human behavior (Bandura, 2003). Self-efficacy is a key component of this theory as it influences how individuals approach and engage in various activities (Bandura, 2001). Bandura proposed that people with high self-efficacy are more likely to set challenging goals, persevere in the face of obstacles, and exhibit greater effort and resilience compared to those with low self-efficacy. Self-efficacy beliefs are developed from a variety of sources, including personal experiences, social persuasion, vicarious learning (observing others), and physiological and emotional states. These beliefs influence individuals’ choices, efforts, and resilience in the face of difficulties (Kanapeckaitė et al., 2022; Bekesiene et al., 2023). Recent research on occupational self-efficacy showed that self-efficacy increases persons’ belief and confidence in performing the tasks, challenges, and stresses associated with their profession (Li et al., 2022; Mei et al., 2022). Additionally, according to the Guo et al. (2017) study, higher occupational self-efficacy can motivate more people to solve work-related problems. Furthermore, researchers have shown that students with lower academic self-efficacy are at increased risk of academic burnout (Yu et al., 2016).

**Figure 1:**
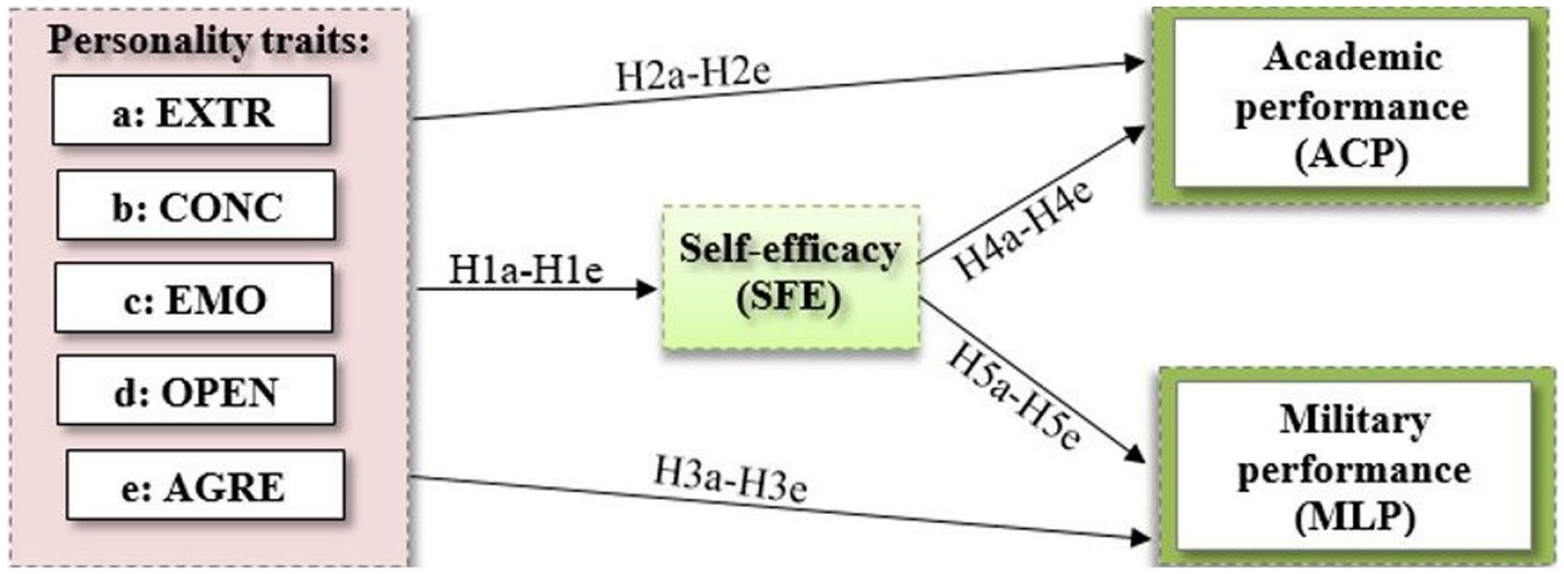
Hypothesized theoretical model characterizes direct and indirect effects of five personality traits (extraversion (a: EXTR), conscientiousness (b: CONC), emotional stability (c: EMO), openness to experience (d: OPEN), and agreeableness (e: AGRE)): direct effects are specified to self-efficacy (hypotheses: H1a – H1e), to academic performance (hypotheses: H2a – H2e), and to military performance (hypotheses: H3a–H3e); the indirect effects (hypotheses H4a-H4e) of personality traits to academic performance (ACP) through self-efficacy (SFE); and hypotheses H5a-H5e are specified to test the indirect effects of personality traits to military performance (MLP) through self-efficacy (SFE).

Taking into account the existing literature and theoretical assumptions, direct pathways were hypothesized from five personality traits to self-efficacy, academic, and military performance in the proposed model (see Figure 1):

Direct effect of five personality traits on self-efficacy:

*H1a-H1e*: Personality traits extraversion (H1a: EXTR), conscientiousness (H1b: CONC), emotional stability (H1c: EMO), openness to experience (H1d: OPEN), and agreeableness (H1e: AGRE) have an effect on self-efficacy.

Direct effect of five personality traits on academic performance:

*H2a-H2e*: Personality traits extraversion (H1a: EXTR), conscientiousness (H1b: CONC), emotional stability (H1c: EMO), openness to experience (H1d: OPEN), and agreeableness (H1e: AGRE) have an effect on academic performance (ACP).

Direct effect of five personality traits on military performance:

*H3a-H3e*: Personality traits extraversion (H3a: EXTR), conscientiousness (H3b: CONC), emotional stability (H3c: EMO), openness to experience (H3d: OPEN), and agreeableness (H3e: AGRE) have an effect on military performance (MLP).

### 2.2. Mediating effect of self-efficacy on academic and military performance

Bandura’s social cognitive theory and the concept of self-efficacy have been widely applied in various fields, including psychology, education, health, and organizational behavior, to understand and enhance human performance and well-being. The available literature suggests that personality factors and self-efficacy are consistently associated with each other (Ahmadi et al., 2023). Additionally, a recently conducted study confirmed that personality factors and self-efficacy can help improve student performance by manipulating their interest, goal line, and learning are predominantly considered predictors rather than consequences of academic performance (Wang et al., 2023). Previous research conducted has shown that the relationship between personality traits and student performance may be mediated by self-efficacy. Di Giunta et al. (2013) showed that self-efficacy can play an intermediate role in the relationship between conscientiousness and openness and academic achievement. Furthermore, Caprara et al. (2011) confirmed mediation of self-efficacy between conscientiousness and academic achievements in the longitudinal study. Additionally, the results of the Hayat et al. (2020) study showed that individual differences in personality traits directly and indirectly play an essential role, through self-efficacy, in contributing to student academic performance; the positive indirect effect of conscientiousness and openness, and the negative indirect effect of neuroticism on academic performance through self-efficacy was confirmed.

Building and maintaining self-efficacy in the military often involves a combination of training, experience, positive reinforcement, mentorship, and providing opportunities for individuals to demonstrate and develop their skills (McCrory et al., 2013). By fostering a sense of self-efficacy among service members, military organizations can improve individual and collective effectiveness, as well as promote the overall well-being and resilience of their personnel (Bekesiene et al., 2023). Considering meaning in military performance as a complex individual construct, indirect pathways linking it to personality dimensions become significant. Following Fosse et al. (2015) it is not enough to take into account only personality traits characteristics as representations to evaluate if the performance of the cadets is suitable, and self-efficacy must also be included. It is especially true in the context of military performance, which refers to an individual’s belief in their own abilities to successfully perform specific tasks or achieve specific goals. Having a strong sense of self-efficacy in the military can lead to improved decision-making, adaptability, and overall mission success (Bekesiene et al., 2022; Lucier-Greer et al., 2022; Qiu et al., 2023). It helps individuals to believe in their own abilities to perform their duties and contribute effectively to the team or unit. In contrast, low self-efficacy can lead to self-doubt, decreased motivation, and potentially impact performance and mission outcomes. Therefore, professional self-efficacy may play an important role in the academic and military achievements of the cadets. However, there is little evidence to support the relationship between professional self-efficacy and levels of academic and military burnout among military academy students.

Taking into account the aspects of studies in the military academy, it can be expected that the military performance of the cadets was strengthened by the individual’s belief in their own abilities to successfully perform specific tasks or achieve specific goals. Therefore, the indirect effect of five personality traits on the academic and military performance of cadets through self-efficacy was hypothesized:

Mediation effect of self-efficacy on academic performance:

*H4a-H4e*: Self-efficacy (SFE) positively mediates the relationship between personality traits extraversion (H4a: EXTR), conscientiousness (H4b: CONC), emotional stability (H4c: EMO), openness to experience (H4d: OPEN), agreeableness (H4e: AGRE) and academic performance (ACP).

Mediation effect of self-efficacy on academic performance:

*H5a-H5e*: Self-efficacy (SFE) positively mediates the relationship between personality traits extraversion (H5a: EXTR), conscientiousness (H5b: CONC), emotional stability (H5c: EMO), openness to experience (H5d: OPEN), agreeableness (H5e: AGRE) and military performance (MLP).

## 3. Research methodology

### 3.1. Design, place of study, and ethical aspects

This study employed a random sampling method. Data were collected through self-reported questionnaires applied digitally (Google Forms) in spring 2023 in Lithuanian Military Academy (LMA). Before filling out the questionnaire, the cadets were informed of the ethical principles of anonymity and confidentiality of the data to be collected. The participation was fully voluntary with no rewards. The sample included 120 cadets attending the 3rd and 4th courses of the LMA. This contained within 80 men (77,5%) and 27 women (22,5%). The average age of the cadets was 21.9 years (±SD = 1.18, range = 20–27 years). Most of the 112 (92,5%) cadets had secondary school education and confirmed they had no previous military experience. In relations of age and military skills, the cadets of Lithuanian Military Academy in general are comparable with those of other NATO nations. Each participant was provided with information about the study; informed consent was obtained from all students before starting the study; their voluntary participation and anonymity were ensured.

### 3.2. Measures

#### 3.2.1. Background factors and control variables

Demographic variables such as gender, age, and level of civilian education level were included in the research questionnaire to represent the background of the study respondents. The achievement level of education was evaluated using a three-point scale: 1 = ‘secondary school’, 2 = ‘unfinished college or university’, 3 = ‘finished college or university’; gender as a categorical dichotomous variable was coded 1 = male and 0 = female; to define the study course of cadets was used codes: 1 = 3rd course, 0 = 4th course; age was a parametric variable measured on an interval scale. Despite the fact that gender, age and study course variables refer to background factors, they may affect the research results (Kusurkar et al., 2010; Paray and Kumar, 2020; Wang et al., 2023). Also, previous research has demonstrated that differences in the prediction of male and female academic success are statistically significant (Mellon et al., 1980), and female students showed a higher course grade average than male students (Conger and Long, 2010). Therefore, the gender, age, and study course variables were included in this study to evaluate for their potential influence.

#### 3.2.2. The Big five personality dimensions

The personality dimensions of the cadets were evaluated using the well-known Big Five Inventory (BFI) developed by Benet-Martínez and John (1998). Used BFI contains 44 items and represents a five-factor model of personality: extraversion (8 items), conscientiousness (9 items), neuroticism (8 items), openness to experience (10 items), and agreeableness (9 items). The BFI items were answered using a 5-point Likert type scale: 1 = ‘I strongly disagree’, 2 = ‘I disagree’, 3 = ‘Undecided’, 4 = ‘I agree’ and 5 = ‘I strongly agree’. The internal consistency was evaluated by Crohnbach’s alfa for each personality dimension: extroversion (*α* = 0.835), conscientiousness (*α* = 0.767), emotional stability (*α* = 0.775), openness to experience (*α* = 0.754), and agreeableness (*α* = 0.712).

#### 3.2.3. Self-efficacy scale

Taking into account the insights of self-efficacy theorists (Urdan and Pajares, 2006), it is not appropriate to use a universal or more general scale for measuring self-efficacy as cadets should evaluate their own effectiveness taking into account their accumulated experience of the environment in which military training and academic studies take place (Boe et al., 2018). In this sense, the questionnaire that covered areas of self-efficacy related to studying at the military academy was used. Despite the fact that Buch et al. (2015) validated statements for military academic self-efficacy evaluation, these statements were adapted to measure the perceived competence of cadets to achieve the military academy context. The seven questions were presented for the respondents to report their self-efficacy on a 5-point scale ranging from 1 = ‘totally disagree’ to 5 = ‘totally agree’. According to Nunnally (1978) suggestions, it can be stated that previous studies (Buch, et al., 2015; Fosse et al., 2015) confirmed high internal consistency of this scale, the indicated Cronbach alphas were above 0.70 in both studies (ranged from 0.83 to 0.89). Also, it can be mentioned that in the current study, the adapted self-efficacy scale showed respectable internal consistency, the Cronbach’s α of 0.884.

#### 3.2.4. Academic performance evaluation

Cadets’ academic performance (ACP) was measured through academic achievements. Studies in the military academy include several courses that represent 210 ECTS-credit points for the three-and-a-half year. The academic achievements of the cadets were evaluated by the Grade Point Average (GPA) as an indicator of their academic performance. The GPA is a number representing the average value of the final grades accumulated received after exams such as English, military leadership and strategy were used as cadet performance indicators. Despite the fact that the academic achievement values were received on a ten-point scale from the military academy learning center, for this study they were transformed to a five-point scale. In this study, the GPA assessment ranges from 1 to 5, where the five represent the highest achievement (evaluation of 9–10 points) and the best cadet’s performance grade.

#### 3.2.5. Military performance evaluation

Cadets’ military performance (MLP) was assessed using instructor ratings. The collected score highlights the overall impression of the improvement of the cadets’ military capabilities during 3 years (4th course) and two and a half years (3rd course). The assessment is accepted by two instructors. The grades which have been used involve items from the subsequent 10 domains: basics of first aid, preparation of equipment, recognition of topographic signs and object coordinates on the map, knowledge about weapons and shooting achievements, cooperation/communication, leadership, and coping. Responsible instructors evaluate cadets on a 5-point Likert-type scale from 1 to 5: 1 = ‘below average’, 2 = ‘slightly below average’, 3 = ‘average’, 4 = ‘slightly above average’, and 5 = ‘above average’. The average score of the included domains was used for the analysis. These grades give an overall impression of the cadets’ military capability. The military performance scale showed high internal consistency the Cronbach’s α of 0.942.

### 3.3. Methods of statistical analysis

Before all statistical analyzes the sample size evaluation test was conducted by using well-known G∗Power v3.1.9.4 package. The F tests for Linear multiple regression was based on five predictors with a significance level of 0.05, power of 0.90, and effect size of 0.15. was defined that to reach statistical power is required a sample size of 116. Additionally, the post-hoc test was performed to compute achieved power with significance level of 0.05, the sample size of 120 (a valid data set was used), and effect size of 0.15; it helped to reveal that even with the sample size of 114 can be reached the statistical power equal to 0.9129. Statistical studies were performed using IBM SPSS Statistics 29v and SPSS AMOS 29v. The individual level of analysis was used for collected demographic data and study constructs: (i) extraversion of five personality traits (EXTR), conscientiousness (CONC), emotional stability (EMO), openness to experience (OPEN), and agreeableness (AGRE); (ii) self-efficacy (SFE); (iii) academic performance (ACP) and (iv) military performance (MLP). The descriptive statistic was used to evaluate the statistical means and standard deviations (M and ± SD) of the variables, and then the Pearson bivariate correlation procedure was used to evaluate the relationships. To reduce the influence of common method bias in the study were used two different methods: first approach focused on developing instruments that emphasized anonymity and confidentiality of responses; as second approach Harman’s single-factor test was used to investigate the potential variance of the common method (Tehseen et al., 2017). The convergence of variables constructs was evaluated by average variance extracted (AVE) and composite reliability (CR) (Fornell and Larcker, 1981). Also, the discriminant validity and confidence intervals were used to demonstrate discriminant validity between constructs (Tehseen et al., 2017). Structural Equation Modeling Analysis (SEMA) was used to assess the hypothesized model. Before modeling, there was performed a factor analysis to assess the latent constructs and variables. Only then was the modeling process continued with theoretical causal model path analysis (Byrne, 2013). Causal interactions between eight factors were recognized by SEMA. Theorized direct and indirect links among study constructs were tested in specified four models: Model 1 was conducted to test how five personality traits affect self-efficacy (hypotheses: H1a-H1e); using Model 2 were evaluated pathways among five personality traits and academic performance (hypotheses: H2a-H2e); using Model 3 were tested how five personality traits affect military performance (hypotheses: H3a-H3e); and using Model 4, which was extended by three control variables (course, gender, and age), the theorized indirect effects between all constructs were evaluated (hypotheses: H4a-H4e and H5a-H5e). According to the proposed Model 4 design, 11 variables were recognized: three observed endogenous variables (MLP, SFE, ACP); five observed exogenous variables (EXTR, EMO, AGRE, CONC, and OPEN); and three unobserved exogenous variables.

The hypothesized relations between the model constructs were tested using SPSS AMOS 29v, and the coefficient weights were chosen to assess the causal relations by agreeing with suggestions of previous scholars (Bekesiene et al., 2017a,b, 2022; Smaliukienė et al., 2023) who propose a versatile methodology for assessing the suitability of a theoretical model. Therefore, the goodness of fit of the models was estimated by subsequent criteria: (1) the probability statistic of *χ*^2^ likelihood ratio, (2) the Tucker and Lewis Index (TLI), (3) the Comparative Fit Index (CFI), and (4) the Root Mean Square Error of Approximation (RMSEA) with related confidence intervals (CI). Only values greater than 0.95 values of the TLI and CFI indices (Hu and Bentler, 1999) and values lower than 0.08 for the RMSEA measure (Browne and Cudek, 1993) were accepted. Data analysis was conducted, and model parameters were estimated using full information maximum likelihood (Maydeu-Olivares and García-Forero, 2010). The bootstrapping analysis of 5,000 was conducted and confidence acceptance was established at 95% bias-corrected confidence intervals (95% CI). The effects of indirect relationships were considered statistically significant if zero was not included in the 95% bias-corrected CI (Hayes and Scharkow, 2013; Hair, 2019).

## 4. Study results

### 4.1. Preliminary analyzes for scale evaluations

As mentioned previously, descriptive statistical analysis was performed at the individual level and preliminary information on research variables was collected. Furthermore, the relationships between the study variables were evaluated by calculating Pearson’s bivariate correlation coefficients. In addition, the convergent and discriminant validity of the designed constructs were estimated. These results are presented in Table 1.

**Table 1:** The descriptive statistics, discriminant validity, and Pearson’s correlations between the study variables.

Variables	Descriptive statistics			Correlations							
	M	±SD	Age	1	2	3	4	5	6	7	8
Control variables											
Course of study	--	--									
Gender	--	--									
Age	21.86	1.18	**--**								
Independent variables											
1: Extraversion	4.42	0.98	−0.191*	(0.835)							
2: Conscientiousness	4.80	0.54	0.117	0.140	(0,767)						
3: Emotional stability	4.41	0.96	−0.082	−0.227*	0.157*	(0.775)					
4: Openness to experience	4.37	0.99	0.014	−0.140	0.173*	0.287**	(0.754)				
5: Agreeableness	4.36	0.97	−0.025	−0.069	−0.086	0.303**	0.052	(0.712)			
6: Self-efficacy	3.74	0.88	−0.015	0.227*	0.302**	0.026	0.243**	−0.082	(0.884)		
Dependent variables											
7: Academic performance	3.87	0.62	0.045	0.313**	0.419**	0.110	0.171*	−0.073	0.560**	--	
8: Military performance	4.17	0.71	0.136*	0.250**	0.363**	0.105	0.187*	−0.039	0.525**	0.518**	(0.942)

Pearson’s correlation is significant at: *p < 0.05 level (2-tailed); **p < 0.01 level (2-tailed). M-mean; ± SD-standard deviation. Cronbach’s alpha coefficients are shown in parentheses on the diagonal.

The correlation coefficients (see Table 1) indicated that academic performance is positive associated with military performance (ACP&MLP, *r* = 0.518, *p* < 0.01), and self-efficacy (ACP&SFE, *r* = 0.560, *p* < 0.01). Military performance also indicated positive and highly significant relationships with self-efficacy (MLP&SFE, *r* = 0.525, p < 0.01). The Big Five personality traits such as extraversion (EXTR), conscientiousness (CONC) and openness to experience (OPEN) had statistically significant relations with: (i) self-efficacy (EXTR&SFE, *r* = 0.227, *p* < 0.05; CONC&SFE, *r* = 0.302, *p* < 0.01; OPEN&SFE, *r* = 0.243, *p* < 0.01); (ii) academic performance (EXTR&ACP, *r* = 0.313, *p* < 0.01; CONC&ACP, *r* = 0.419, p < 0.01; OPEN&ACP, *r* = 0.171, *p* < 0.05); and (iii) military performance (EXTR&ACP, *r* = 0.313, *p* < 0.01; CONC&ACP, *r* = 0.419, *p* < 0.01; OPEN&ACP, *r* = 0.171, *p* < 0.05). Although personality traits such as emotional stability and agreeability did not show the relationships with self-efficacy, academic or military performance. Finally, age as one of the control variables was positively correlated with military performance (Age&MLP, *r* = 0.136, *p* < 0.05).

Harman’s single-factor test was conducted. The findings of exploratory factor analysis (EFA) accounted for 67.57% of the total variance for greater than one factor, and 30.54% of the covariance between the measures was indicated for a single factor. Furthermore, to demonstrate the reliability of the study constructs, composite reliability (CR) values were computed. The results of statistical analysis let us establish the adequacy of eight constructs, the CR values ranged from 0.903 to 0.932. Furthermore, by evaluating the average variance extracted (AVE) the discriminant validity of the study constructs was tested. The get results showed that AVE values of constructs ranged from 0.646 to 0.774 are in an acceptable interval and meet the requirements (Fornell and Larcker, 1981). The discussed values are presented in Table 1.

### 4.2. Hypotheses testing results

Modeling analysis was performed using IBM AMOS 29v software. Confirmatory factor analysis was used to test the theorized links among constructs in specified models. Model 1 tested how five personality traits affect self-efficacy (hypotheses: H1a-H1e); Model 2 evaluated pathways among five personality traits and academic performance (hypotheses: H2a-H2e); and Model 3 tested how five personality traits affect military performance (hypotheses: H3a-H3e). Finally, the control variables (course, gender and age) were included and indirect effects theorized (hypotheses: H4a-H4e and H5a-H5e) between all constructs were evaluated by Model 4. The goodness-of-fit of theorized models was assessed by SEMA results.

#### 4.2.1. Direct-effects evaluation

First, the direct effects of five personality traits extraversion (EXTR), conscientiousness (CONC), emotional stability (EMO), openness to experience (OPEN) and agreeableness (AGRE) to self-efficacy (SFE, H1a-H1e, Model 1), academic performance (ACP, H2a-H2e, Model 2), and military performance (MLP, H3a-H3e, Model 3) were estimated.

The results of a six-factor Model 1, the direct effects of five personality traits on SFE indicated a good fit to the data [*χ*^2^ = 4.044 (df = 4, *p* = 0.40), CFI = 0.999; NFI = 0.941; TLI = 0.997; RMSEA = 0.010, 90% CI: 0.00–0.139; and PCLOSE = 0.546]. Specifically, the *χ*^2^ test of exact fit test 2 was statistically significant and the CFI was higher than the suggested threshold value of 0.90 (Hu and Bentler, 1999), the close fit RMSEA test was well below the threshold of 0.08 (Hair, 2019). Furthermore, it was found that three of the personality traits have a significant, positive, and direct influence on self-efficacy (EXTR → SFE for H1a: *γ* = 0.221, *p* = 0.010; CONC SFE for H1b: *γ* = 0.225, *p* = 0.009; OPEN SFE for H1d: *γ* = 0.239, *p* = 0.006). Thus, hypotheses for the direct effects of personality traits on self-efficacy (SFE) were accepted only for three personality dimensions: extraversion (H1a: EXTR), conscientiousness (H1b: CONC), and openness to experience (H1d: OPEN) (see Model 1, Table 2).

**Table 2:** The direct effects of personality traits evaluated by SEM analysis.

Explanation		Coeff.γ	S.E.	St. Coeff.γ	C.R.	*p*	LLCI	ULCI
Hypotheses H1a-H1e	Model 1							
	EXTR →SFE	0.151	0.058	0.221	2.584	0.010	0.041	0.378
	CONC → SFE	0.188	0.072	0.225	2.595	0.009	0.027	0.406
	EMO → SFE	−0.010	0.086	−0.010	−0.111	0.912	−0.189	0.148
	OPEN → SFE	0.186	0.067	0.239	2.770	0.006	0.060	0.389
	AGRE → SFE	−0.051	0.079	−0.056	−0.649	0.516	−0.254	0.128
		Coeff.γ	S.E.	St. Coeff.γ	C.R.	*p*	LLCI	ULCI
Hypotheses H2a-H2e	Model 2 EXTR →ACP	0.145	0.039	0.302	3.751	***	0.094	0.467
	CONC → ACP	0.194	0.048	0.330	4.041	***	0.154	0.484
	EMO → ACP	0.071	0.057	0.108	1.241	0.215	−0.101	0.300
	OPEN → ACP	0.070	0.045	0.127	1.568	0.117	−0.065	0.296
	AGRE → ACP	−0.040	0.053	−0.063	−0.767	0.443	−0.231	0.108
		Coeff.γ	S.E.	St. Coeff.γ	C.R.	*p*	LLCI	ULCI
Hypotheses H3a-H3e	Model 3 EXTR →MLP	0.135	0.046	0.246	2.915	0.004	0.064	0.410
	CONC → MLP	0.192	0.058	0.285	3.342	***	0.069	0.481
	EMO → MLP	0.062	0.069	0.082	0.898	0.369	−0.098	0.262
	OPEN → MLP	0.094	0.054	0.149	1.749	0.049	0.025	0.315
	AGRE → MLP	−0.023	0.063	−0.031	−0.357	0.721	−0.193	0.124

extraversion (EXTR), conscientiousness (CONC), emotional stability (EMO), openness to experience (OPEN) and agreeableness (AGRE). Self-efficacy (SFE), academic performance (ACP), military performance (MLP). C.R., Critical ratio for regression weight; S.E., Standard error of regression weight; *p*, probability of getting a critical ratio. LLCI, lower limit of 95% CI; ULCI, upper limit of 95% CI; bootstrap sample size = 5000.

Furthermore, Model 2 was conducted to test the direct effects of five personality traits on ACP. The results indicated a good fit to the data [*χ*^2^ = 4.044 (df = 4, *p* = 0.40), CFI = 0.999; NFI = 0.949; TLI = 0.997; RMSEA = 0.010, 90% CI: 0.00–0.139; and PCLOSE = 0.546]. It was established that two of the personality traits have a significant, positive, and direct influence on academic performance (EXTR → ACP for H2a: *γ* = 0.302, *p* < 0.001; CONC ACP for H1b: *γ* = 0.330, *p* < 0.001). Thus, hypotheses for the direct effects of two personality traits on academic performance (ACP) were accepted: extraversion (H2a: EXTR) and conscientiousness (H2b: CONC). Detailed information about Model 2 is presented in Table 2.

Lastly, the six-factor Model 3 was developed to evaluate the direct effects of five personality traits on MLP was worked out. Model 3 indicated a good fit to the data [*χ*^2^ = 4.044 (df = 4, *p* = 0.40), CFI = 0.999; NFI = 0.941; TLI = 0.997; RMSEA = 0.010, 90% CI: 0.00–0.139; and PCLOSE = 0.546]. Additionally, three of the personality traits were recognized to have a significant, positive, and direct influence on military performance (EXTR → MLP for H3a: *γ* = 0.246, *p* = 0.004; CONC MLP for H3b: *γ* = 0.285, *p* < 0.001; OPEN MLP for H3d: *γ* = 0.149, *p* = 0.049). Therefore, hypotheses for direct effects of personality traits on military performance (MLP) were accepted only for three personality dimensions: extraversion (H3a: EXTR), conscientiousness (H3b: CONC), and openness to experience (H3d: OPEN) (see Model 3, Table 2).

#### 4.2.2. Mediation effect of self-efficacy

Following this research methodology, Model 4 was extended by three control variables (course, gender, and age) and theorized indirect effects (hypotheses: H4a-H4e and H5a-H5e) were tested. Following the hypotheses H5a-H5e, the mediating relationships of self-efficacy (SFE) were tested between five personality traits (EXTR, CONC, EMO, OPEN, and AGRE) and academic performance (ACP). Also, the hypotheses H5a-H5e between personality traits and military performance were evaluated.

**Figure 2:**
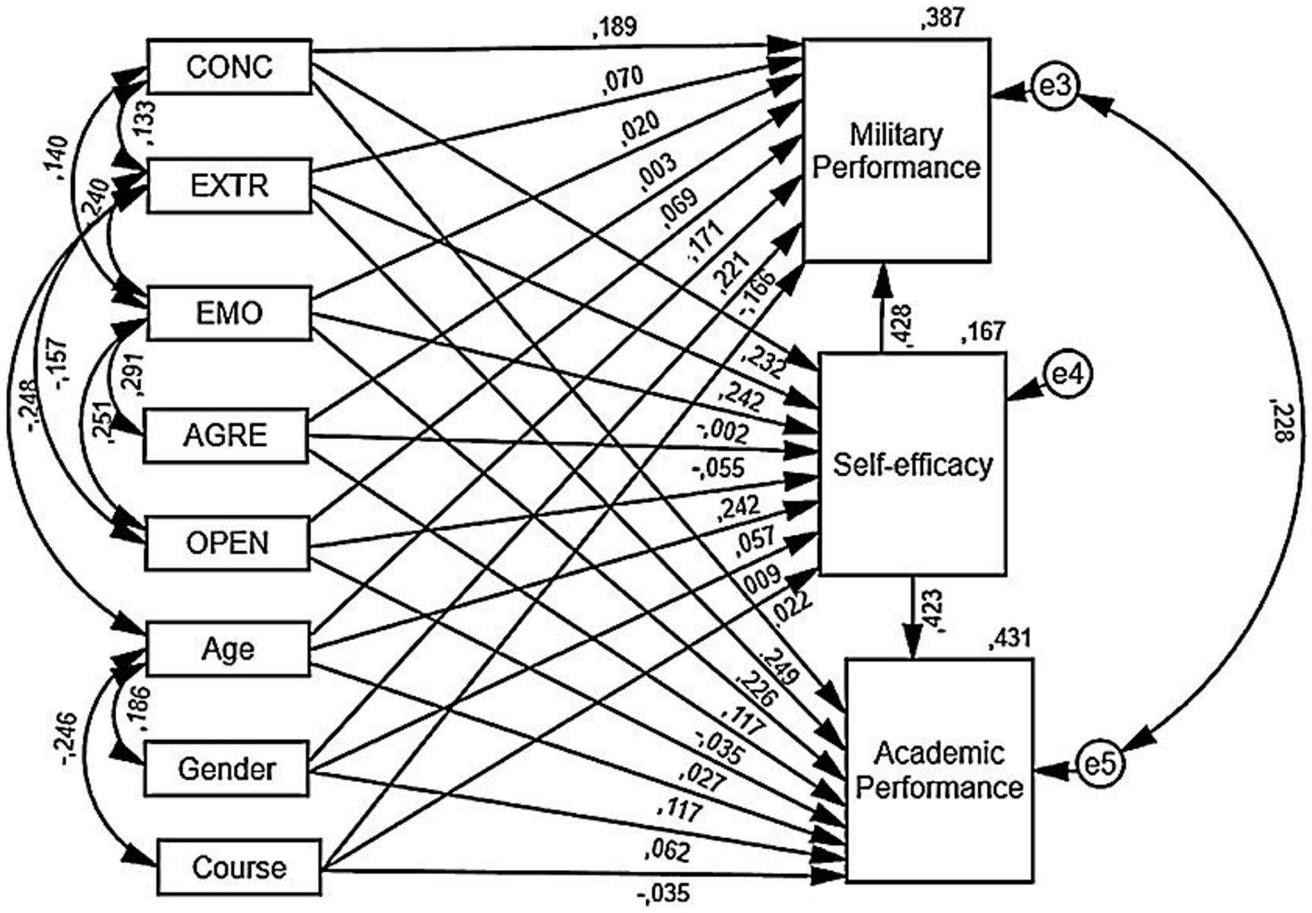
Graphical representation of the causal relations between personality dimensions, military, and academic performance with self-efficacy mediation and effect of control variables (age, gender, and course) in Model 4 [*χ*^2^ (19) = 18,703, *p* = 0.476; RMSEA = 0.010, 90% CI: 0.00–0.079; CFI = 1.000, NFI = 0.921 and PCLOSE = 0.767]. The abbreviations are used to represent five personality traits extraversion (EXTR), conscientiousness (CONC), emotional stability (EMO), openness to experience (OPEN) and agreeableness (AGRE).

The analysis carried out showed that the designed Model 4 indicated good consistency with the collected data [*χ*^2^ (19) = 18,703, *p* = 0.476; RMSEA = 0.010, 90% CI: 0.00–0.079; CFI = 1.000, NFI = 0.921]. Graphical representation of the causal relationships between personality dimensions, military and academic performance with self-efficacy mediation and the effect of the control variables (age, gender, and course) is presented in Figure 2.

The detailed study results presented in Table 3 confirmed that in Model 4 three personality traits were positively and significantly related to self-efficacy (SFE): extraversion (H1a: EXTR → SFE, *γ* = 0.242, *p* < 0.01), conscientiousness (H1b: CONC → SFE, *γ* = 0.232, *p* < 0.01), and openness to experience (H1d: OPEN → SFE, *γ* = 0.221, *p* < 0.01). Additionally, self-efficacy was positively related to academic performance (SFE → ACP, *γ* = 0.423, *p* < 0.001) and military performance (SFE → MLP, *γ* = 0.428, *p* < 0.001). Approximately 43% of the variance in academic performance was accounted for by the predictors (*R*^2^ = 0.43, Table 3) and 39% of the variance in military performance (*R*^2^ = 0.39, Table 3).

**Table 3:** The direct and indirect standardized effects evaluated in Model 4 using SEM analysis.

Predictor variables	Predicted variable								
	Self-efficacy (SFE)	Academic Performance (ACP)				Military Performance (MLP)			
	Direct	Direct	Indirect Effect			Direct	Indirect Effect		
	St. Estim. γ	St. Estim. γ	St. Estim. γ	95%CI		St. Estim. γ	St. Estim. γ	95%CI	
				LLCI	ULCI			LLCI	ULCI
Control variables									
Course_3rd	0.022	−0.035				−0.166*	0.009	−0.068	0.087
Gender	0.009	0.062				0.221**	0.004	−0.071	0.087
Age	0.057	0.117				0.171*	0.024	0.071	0.227
Independent variables									
Extraversion	0.242**	0.226**	0.102**	0.029	0.197	0.070	0.104**	0.025	0.259
Conscientiousness	0.232**	0.249***	0.098*	0.019	0.197	0.189*	0.099*	0.019	0.207
Emotional stability	−0.002	0.117				0.020			
Openness to experience	0.221**	0.027	0.101**	0.028	0.196	0.069	0.100**	0.028	0.197
Agreeableness	−0.055	−0.035				0.003			
Mediator									
Self-efficacy		0.423***				0.428***			
Predictors explain output variance (*R*^2^)	0.17	0.43				0.39			

St. Estim. *γ* – standardized estimations. Significance at: *p < 0.05; **p < 0.01; ***p < 0.001 (2-tailed). *R*^2^ – estimate of squared multiple correlation. Gender codes: 1 = male, 0 = female. Course codes: 1 = 3rd course, 0 = 4th course. LLCI = lower limit of 95% CI; ULCI = upper limit of 95% CI; bootstrap sample size = 5000.

Furthermore, the results of the modeling of the indirect effect of personality traits on academic performance (ACP) and military performance (MLP) were evaluated using the bias-corrected percentile bootstrap approach based on 5,000 bootstrap samples estimated with a 95% confidence interval. The conducted analysis let to establish positive and significant indirect relationships from extraversion [H4a: EXTR × SFE → ACP, standardized effect = 0.102, *p* < 0.01, 95% CI = (0.029, 0.197)], conscientiousness [H4b: CONC × SFE → ACP, standardized effect = 0.098, *p* < 0.05, 95% CI = (0.019, 0.197)], and openness to experience [H4d: OPEN × SFE → ACP, standardized effect = 0.101, *p* < 0.01, 95% CI = (0.028, 0.196)] on academic performance (ACP). Furthermore, the weighted indirect effect through self-efficacy (SFE) on military performance (MLP) was confirmed: extraversion [H5a: EXTR × SFE MLP, standardized effect = 0.104, *p* < 0.01, 95% CI = (0.025, 0.209)], conscientiousness [H5b: CONC × SFE → ACP, standardized effect = 0.099, *p* < 0.05, 95% CI = (0.019, 0.207)], and openness to experience [H5d: OPEN × SFE → ACP, standardized effect = 0.104, *p* < 0.01, 95% CI = (0.028, 0.197)]. Following bootstrap with the 5,000 sample size test and 95% CI with no zero, the significant indirect effects were verified only for personality traits such as EXTR, CONC, and OPEN. Therefore, hypotheses H4a, H4b, H4d, H5a, H5b, and H5d are confirmed (see Table 3).

While the results of the modeling showed that SEF mediation for academic performance (ACP) can be classified as ‘partial mediation’ (Baron and Kenny, 1986) for conscientiousness (CONC→ ACP, *γ* = 0.249, *p* < 0.001), extraversion (EXTR→ ACP, *γ* = 0.226, *p* < 0.01), and military performance (CONC→ MLP, *γ* = 0.189, *p* < 0.05). The different situation appears with SEF mediation effects when the effect of personality traits on military performance was evaluated. Extraversion personality traits (EXTR) and openness to experience (OPEN) did not have significant direct relations with military performance and were identified as positive and significant only indirect effects (EXTR→ MLP, *γ* = 0.104, *p* < 0.01 and OPEN MLP, *γ* = 0.100, *p* < 0.01), accordingly the SEF fully mediates EXTR and OPEN personality traits to military performance.

**Figure 3:**
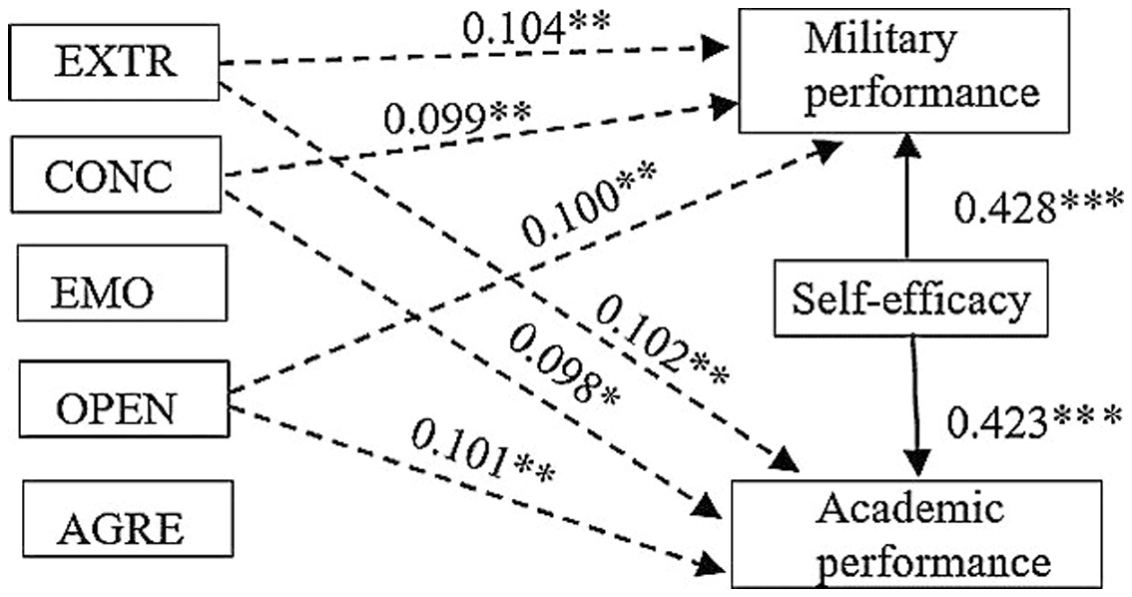
Graphical representation of indirect effect among personality dimensions’, military and academic performance when mediation effect of self-efficacy is taken into account in Model 4 [*χ*^2^ (19) = 18,703, *p* = 0.476; RMSEA = 0.010, 90% CI: 0.00–0.079; CFI = 1.000, NFI = 0.921 and PCLOSE = 0.767]. Dashed arrows illustrate identified significant indirect relationships between three dimensions of personality (conscientiousness (CONC), extraversion (EXTR), openness to experience (OPEN)), military and academic performance. The straight arrows characterize direct effect of self-efficacy to military and academic performance. The standardized path coefficients with significance indicator (*p < 0.05; **p < 0.01; ***p < 0.001) are marked up close to the arrows.

Accordingly, the hypotheses presented H4a-H4e ‘Self-efficacy (SFE) positively mediates the relationship between personality traits for academic performance’ can be partially confirmed for extraversion (H4a), conscientiousness (H4b), and openness to experience (H4d). Different situations appear when H5a-H5e ‘Self-efficacy (SFE) positively mediates the relationship between personality traits for military performance’. Self-efficacy fully mediates personality traits such as extraversion (H5a) and openness to experience (H5d), but also only partially mediates conscientiousness (H4b). The simplified modeling results are presented in Figure 3 and all estimates of Model 4 are reported in Table 3.

Finally, the results of this study confirmed that only military performance showed the statistically significant relationships with control variables: gender (*γ* = 0.221, *p* < 0.01, Table 3), course (*γ* = −0.116, *p* < 0.05, Table 3), and age (*γ* = 0.171, *p* < 0.05, Table 3).

## 5. Discussions

This research focused on evaluating direct and indirect relationships among the dimensions of the Five Big Five personality traits such as ‘Extraversion’, ‘Neuroticism’, ‘Openness’, ‘Conscientiousness’ and ‘Agreeableness’, academic and military performance through self-efficacy. Previous studies mostly focused on the conscientiousness dimension as it is generally associated with disciplined behavior, attention to detail, and a strong work ethic (McCrae and Costa, 1999). Similarly, individuals with conscientiousness need to be responsible, organized, and reliable. So, in the military, these people tend to perform well as they are likely to follow rules and procedures, complete tasks thoroughly, and show commitment to their duties (Fosse et al., 2015). Current modeling analysis conducted confirmed the partly mediation of self-efficacy between conscientiousness and both dependent variables academic performance and military performance. These findings are in line with previous research conclusions, where it was confirmed that conscientiousness is often positively correlated with job performance, reliability, and adherence to military protocols (Barrick and Mount, 1991; Barrick et al., 2001; Judge et al., 2002; Fosse et al., 2015).

Get results also propose that self-efficacy partly mediated relationships between academic performance and extraversion personality trait which is characterized as outgoing, energetic, and sociable behavior. Furthermore, this research proved that self-efficacy can completely mediate the relations between extraversion and military performance. This finding can be explained, that in military settings, extraverted individuals may excel in roles that involve teamwork, leadership, and social interaction. They often display strong communication skills, confidence, and assertiveness, which can be valuable in certain positions, such as leading troops or participating in public relations activities (Judge et al., 2002).

Additionally, this study confirmed that self-efficacy completely mediated the relations between openness to the experience personality dimension and academic performance, as well as between openness to the experience dimension and military performance. In a military context, high levels of openness to experience personality can lead to innovative thinking, adaptability, and the willingness to explore new strategies. It may be beneficial in situations that require flexibility, problem solving, and creative decision-making (Jackson et al., 2012).

Furthermore, the findings of the current study proved that self-efficacy, a significant aspect of the social cognitive theory developed by the psychologist Albert Bandura (1999), can indeed affect the development of behavior and regulate personality traits. According to Bandura (2012), beliefs about self-efficacy influence the choices people make, the amount of effort they put into tasks, their resilience in the face of obstacles, and their overall level of motivation. Overall, self-efficacy plays a crucial role in shaping behavior and influencing the development of personality traits. By improving self-efficacy beliefs, people can improve their performance, increase motivation, and cultivate positive behavioral patterns that contribute to personal growth and success. Recent studies have shown that individual self-perception and experiences can control their achievements (Mohebi and Bailey, 2020).

Particular attention in this study was paid to the influence of self-efficacy, as a critical factor that can significantly shape students’ experiences and achievements in the field of education (Lievens et al., 2009). Although the impact of emotional stability and agreeableness personality traits was expected to be rather small, the results showed a statistically insignificant correlation between these factors and the complete performance of the cadets. Although based on previous research, it was expected that the effects of emotional stability and agreeableness personality traits on cadet performance would be relatively small, but the results of the study did not show a significant correlation between these factors and the academic or military performance of the cadets. Therefore, the hypotheses raised regarding personality characteristics, emotional stability and agreeableness, were not confirmed. These results are in line with previous findings that emotional stability is generally not a significant predictor of academic achievement (Judge et al., 2002; Robbins et al., 2004; Guntern et al., 2017), and the impact of agreeableness on academic achievement was rather small and not always consistent between samples (Poropat, 2009).

In this context, our study extends existing research by showing a mediation effect of self-efficacy on three personality traits of cadets. In addition, it was found that the positive mediating effects of self-efficacy on academic performance and military performance are high statistically significant for two personality dimensions, such as extraversion and openness to experience. More specifically, this research included control variables such as gender, study course and age. The findings indicate that these control variables have statistically significant relations only with military performance. The age of the cadets was positively and statistically significant with respect to military performance. The gender was coded 1 = ‘male’ and 0 = ‘female’, and the results showed that the cadets of the category ‘male’ are statistically significant higher in military performance, then ‘females’. Furthermore, these results acknowledge that the 3rd year cadets are statistically significantly worse in military performance than those of the 4th year study. These findings are in line with similar studies (Myrseth et al., 2018; Kanapeckaitė et al., 2022; Nordmo et al., 2022) that support the perceptions that building a strong foundation of knowledge, skills, and competencies, as well as fostering a growth mindset and a supportive learning environment, can contribute to enhancing self-efficacy and, consequently, improving not only academic performance among cadets, but also the higher military performance as was presented by male cadets from the 4th course.

In summary, the theoretical implication of this research contributes to expanding the understanding of personality trait theory and social cognitive theory in the military context. First, the extended knowledge of the getting results on how mediation effects applies to the military performance of cadets. Second, despite the fact that previous studies focused only on the conscientiousness dimension (Caprara et al., 2011; Di Giunta et al., 2013; Fosse et al., 2015), the current modeling analysis contained within five dimensions of personality traits. Third, the analysis performed proved the correspondence of the proposed theoretical model with the data, so it can be said that the presented study clarifies and complements the existing studies.

In addition, it is important to note that the use of personality tests in a military context has its complexities and challenges, which include ensuring the validity and reliability of such tests, avoiding bias, and accurately interpreting the results. In particular, the development and implementation of personality tests require ongoing research and validation to ensure that they are effective in predicting behavior and performance. Taking into account that specific personality traits of individuals can have implications for their suitability for certain military roles and responsibilities, the personality tests are indeed used and researched within the armed forces of various countries such as Norway, Australia (McCormack and Mellor, 2002), France (Congard et al., 2012) and United States (Chappelle et al., 2010). Overall, while cognitive abilities play a significant role in military performance, personality tests contribute to a more comprehensive understanding of an individual’s traits and characteristics, helping military organizations make informed decisions about recruitment, training, leadership development, and team dynamics. So, these findings may have practical implications not only for predicting and improving academic and military performance, but also for improving personality tests design. Moreover, these findings can help military academy instructors use the results to improve and regulate educational programs by focusing on ways to improve the self-efficacy of the cadets and, as a result, their performance.

Research carried out has limitations that must be considered when interpreting the results presented. A general limitation of the results is that the instrument used to assess self-efficacy strengths was evaluated by self-evaluation and self-perception, and contextual factors of the geopolitical situation during this study may have influenced the general mood and self-esteem of the cadets. Another limitation is that a small group of women was included in the comparison of personal traits between man and women groups. Also, it must be mentioned that while personality traits and self-efficacy beliefs can provide valuable insights into predicting cadets’ academic or military performance, they are not the sole determinants. Military performance is a complex outcome influenced by numerous factors, including training, experience, teamwork, leadership, and situational demands. Other factors such as intelligence, study habits, social support, and environmental factors also contribute to the results of a cadet. One more weakness is that comparing cadets who study in the third year and the last year in the military academy also can raise limitations because self-efficacy is not a fixed trait and can be developed and strengthened over time by promoting a growth mindset and providing opportunities for learning and skill development. Military organizations can help their personnel cultivate and maintain high levels of self-efficacy, thereby enhancing their performance and well-being in challenging situations. Considering the fact that the research was cross-sectional, it is not possible to unambiguously evaluate the identified differences between the third and fourth year cadets’ performance since the time spent studying and the time spent in military training are totally different. Therefore, it would be appropriate for future hypotheses to be supported by a longitudinal study.

In conclusion, the study conducted extends the understanding of the relationships between five personality dimensions, military and academic performance. The insights of this study can be seen as a beneficial suggestion to foster strong self-efficacy in military professionals, training and development programs which can focus on building competence, providing opportunities for mastery experiences, offering constructive feedback and recognition, and promoting a supportive and empowering organizational culture.

Identified gender differences in military training achievements encourage the introduction of innovations in military training programs that would help inspire female cadets to pursue an officer’s career, breaking away from traditional gender norms.

Additionally, this study suggests focusing on the personality of the cadets which was identified as beneficial in the armed forces such as United States, Norway, France and others. It seems that personality testing can provide valuable insights into an individual’s character traits, strengths, weaknesses, and overall suitability for specific roles or professions, including military service. So, testing can help assess traits such as leadership potential, resilience, teamwork, adaptability, and integrity, which are crucial in military environments. It is important to note that while personality testing can provide valuable insights, it should be used in conjunction with other assessment tools and considerations. The holistic evaluation of cadets should encompass various factors, including physical fitness, aptitude, training performance, and ethical values, to make informed decisions about their suitability for military service and career development.

## Data availability statement

The raw data supporting the conclusions of this article will be made available by the author, without undue reservation.

## Ethics statement

Ethical approval was not required for the study involving humans because the study involved a questionnaire. The study was conducted in accordance with the local legislation and institutional requirements. Written informed consent for participation was not required from the participants in accordance with the national legislation and institutional requirements because the study involved a questionnaire and consent is implied by participation in the study.

## Author contributions

SB: Conceptualization, Data curation, Formal Analysis, Funding acquisition, Investigation, Methodology, Project administration, Resources, Software, Supervision, Validation, Visualization, Writing – original draft, Writing – review & editing.
